# Twenty-One Years' Financial Progress

**Published:** 1919-09-20

**Authors:** 


### THE VOLUNTARY HOSPITAL ESSENTIAL TO PUBLIC SAFETY.

### II. Twenty-one Years' Financial Progress.

### THE MINISTRY OF HEALTH CREATES NEW STATE HOSPITALS?

On page 589 of The Hospital of 13th inst., we dealt principally with the surplus income of 2,002 voluntary charities and 846 hospitals, comparing five consecutive years, *i.e.*, the yield in surplus income for each of the years 1913 to 1917, both inclusive. To-day, in view of certain questions which have been asked *re* Government grants, it may further interest our readers to give the figures showing the increase in income and in expenditure during twenty-one years from 1896 to 1917 inclusive. In 1896 the total income from voluntary charities, of which particulars are given in "Burdett's Hospitals and Charities," amounted to £8,011,000. In 1917, the income of the charities had increased to £16,709,000, that is to say, it had more than doubled. Amongst the groups of charities included in these figures were hospitals, where the income increased from £2,528,000 in 1896 to £6,385,000 in 1917, or by upwards of 150 per cent, inclusive of the large amounts received from the War Office in respect of military patients. The income of Missionary Societies increased from £2,413,000 to £3,725,000, or roughly 50 per cent, whilst the income of orphanages and other homes and charities increased from £2,278,000 to £5,103,000. In the two latter cases some large figures are included or excluded, which are peculiar to war conditions.

It is interesting to compare the increase in the expenditure of the three groups of institutions, with the income of which we have just dealt, for such comparison manifests that it has increased by leaps and bounds. In 1896 the total expenditure was £7,464,000, which increased in 1917 to £15,222,000, that is to say, it had more than doubled. Apart from the exceptional case of orphanages, homes, and charities, the largest proportionate increase in expenditure took place in connection with the hospitals, which grew from £2,292,000 in 1896 to £5,648,000 in 1917, or by 146 per cent. The expenditure of the missionary societies increased from £2,281,000 in 1896 to £3,489,000 in 1917, but this figure must be qualified by the statement that some important data have not been received.

It cannot fail to be of interest to our readers to know that in dealing with the income and expenditure of voluntary charities and the hospitals of the United Kingdom we have followed their audited figures and compared them during the lengthened period of twenty-one consecutive years. The weeping Willies who during many years, and especially of late, have bemoaned the financial troubles of our hospitals often exaggerate. They forget the strenuous and exceptional time through which we have passed, and are still passing, as one effect of the great war. They omit to remember that during part of the war period very many hospitals were accommodating wounded soldiers and sailors, sometimes to the exclusion of civilian patients; they forgot that although payments were made for the accommodation of and for the help rendered to these Service men, the payments seldom, if ever, covered the increased cost incurred by the hospitals for their patriotic action. Yet the aggregate amounts received from this source amounted to a considerable sum, and pessimists, now that peace has come and these payments are rapidly diminishing, bemoan the loss of this source of income as a fatal danger to the maintenance and prosperity of the hospitals affected. In fewer and fewer numbers there are still managers of such hospitals weak enough to wave the flag of closed beds or wards, though, if they know their business, they must be aware that the closing of such hospital beds would mean, in practice, an increase in the cost of the reduced number of beds that remained, and it would in no way prove effective in securing the desired reduction in the total expenditure. How then can any responsible man or woman venture to talk of closing certain of their hospital beds with the hope of impressing on the public a fear which must prove as ill-founded as the attempt made would be discrediting to its propagandists?

One chief aim we have in view in this series of articles is to create a higher moral tone amongst the weaker brethren by demonstrating that successful hospital financial prosperity lies in the employment by those responsible of the most capable administrators. The efficient financial organiser is the man or woman who understands how to make known with exactness the actual wants and needs of the particular hospital, and so secures the certain sympathy and support of small, as well as large, donors. At the present day no experienced friend of a great charity underestimates the value and importance of the small donors everywhere throughout the country. The income derived from this source alone by the voluntary hospitals during, say, the last fifteen years, is one of the most encouraging and remarkable signs of the confidence, interest, and gratitude felt for the voluntary hospitals, by men, women, and children of all classes throughout Great Britain. The extent and power of this gratitude is really too wonderful to attempt to appraise in words.

#### The Ministry of Health and New State Hospitals.

The creation of the Ministry of Health, the pressing duty which devolves upon this new department

The previous article appeared on September 13, p. 589.

**The Voluntary Hospital Essential**—(*continued*).

of the Government to turn every one of the great Poor-Law infirmaries, which have been adequately equipped and run as modern up-to-date hospitals during the war, into State hospitals, cannot be surpassed. There is at the present time a great demand for additional hospital beds on the part of very large numbers of the civilian population up and down the country, whose claims for hospital care have had to stand aside during war-time, and who should now have these advantages placed at their disposal by the conversion of the really efficient Poor-Law infirmaries into State hospitals of the best type possible. If Dr. Addison is really in earnest as the first responsible head of the Ministry of Health, he should certainly not let the year 1919 come to an end without setting up with all due publicity these new and great State hospitals, which are ready to his hand. The Ministry of Health should establish and start them on a prosperous career of blessing and succour to hundreds of thousands of people, who are greatly in need of the accommodation and treatment which these hospitals could then properly render.

The Ministry of Health has many important duties, and amongst them is the making of new departures, including the encouragement everywhere of the extension of the pay system of hospital relief, organised under a system which secures that the rates of payment shall be graded to meet the requirements and means of all types of patients, from the highest to the lowest. We have written much on this question in the past, and have had the privilege of being responsible for the administration of a prosperous pay hospital for nearly forty continuous years. This hospital was established and opened in 1880; the original buildings were reconstructed in 1902, the expenses being paid out of the income derived from the working of the hospital, under a system, whereby the minimum possible sum was charged for the best accommodation at a self-supporting rate, the doctors' fees in every case being paid by arrangement between the patients or their friends and the attending physicians and surgeons. Most, if not all, of the leading members of the profession, in London, at any rate, have had their patients under treatment at this the first pay hospital in the United Kingdom. During the period of nearly forty years, its management and the benefits it has rendered to many types of people constitute a splendid record of good and faithful service, continuously given on a self-supporting basis, at the minimum cost possible, to all patients and their friends who have had the good fortune to become inmates of this pioneer institution. The history of Fitzroy House Home Hospital is a guarantee and an example for every hospital and its managers, to grasp the opportunity now offered, and so enable them to put the finances of their particular hospital on a sound financial basis. This can be done, providing they have the public spirit, energy, and courage to take their share in a new and great departure in connection with the hospital services. These paying wards are urgently called for and would be welcomed by all classes of people throughout the United Kingdom.

Democracy and Hospital Progress.

In the present article we have ventured still further to illuminate and advance the flag of progress in the directions which the democratisation of the present times and the call and needs of the nation demand. In existing circumstances it is incumbent upon us all to face with undaunted hearts and unwavering faith the legacy of stress and strain left us by the war. We have emerged from a terrific ordeal successfully. Why should we dread the future? It is the duty of institutions, and especially of hospitals, as of individuals, to work harder, to organise their efforts more systematically, and adapt themselves to changed or changing conditions. Personally, we have every confidence that our leading hospitals at any rate will rise to the occasion and prove themselves capable of facing every emergency and of finding a practical solution for every difficulty. In any event, the past history of the voluntary hospital and the present outlook do not warrant pessimism.

